# The pancreatic niche inhibits the effectiveness of sunitinib treatment of pancreatic cancer

**DOI:** 10.18632/oncotarget.10199

**Published:** 2016-06-21

**Authors:** Neus Martínez-Bosch, Pedro Enrique Guerrero, Mireia Moreno, Anabel José, Mar Iglesias, Jessica Munné-Collado, Héctor Anta, Joan Gibert, Carlos Alberto Orozco, Judith Vinaixa, Cristina Fillat, Francesc Viñals, Pilar Navarro

**Affiliations:** ^1^ Hospital del Mar Medical Research Institute (IMIM), Barcelona, Spain; ^2^ Biomedical Research Institute August Pi i Sunyer (IDIBAPS), Barcelona, Spain; ^3^ Centro de Investigación Biomédica en Red de Enfermedades Raras (CIBERER), Barcelona, Spain; ^4^ Pathology Service, Hospital del Mar, Barcelona, Spain; ^5^ Institute for Research in Biomedicine (IRB Barcelona), Barcelona, Spain; ^6^ Catalan Institute of Oncology-IDIBELL, Barcelona University, Barcelona, Spain

**Keywords:** sunitinib, pancreatic cancer, PDA, acinar carcinoma, fibrosis

## Abstract

Current treatments for pancreatic ductal adenocarcinoma (PDA) are ineffective, making this the 4^th^ leading cause of cancer deaths. Sunitinib is a broad-spectrum inhibitor of tyrosine kinase receptors mostly known for its anti-angiogenic effects. We tested the therapeutic effects of sunitinib in pancreatic cancer using the *Ela-myc* transgenic mouse model. We showed that *Ela-myc* pancreatic tumors express PDGFR and VEGFR in blood vessels and epithelial cells, rendering these tumors sensitive to sunitinib by more than only its anti-angiogenic activity. However, sunitinib treatment of *Ela-myc* mice with either early or advanced tumor progression had no impact on either survival or tumor burden. Further histopathological characterization of these tumors did not reveal differences in necrosis, cell differentiation, angiogenesis, apoptosis or proliferation. In stark contrast, *in vitro* sunitinib treatment of *Ela-myc–* derived cell lines showed high sensitivity to the drug, with increased apoptosis and reduced proliferation. Correspondingly, subcutaneous tumors generated from these cell lines completely regressed *in vivo* after sunitinib treatments. These data point at the pancreatic tumor microenvironment as the most likely barrier preventing sunitinib treatment efficiency *in vivo.* Combined treatments with drugs that disrupt tumor fibrosis may enhance sunitinib therapeutic effectiveness in pancreatic cancer treatment.

## INTRODUCTION

Pancreatic ductal adenocarcinoma (PDA) is the most common pancreatic tumor (accounting for more than 90% of cases). PDA is aggressive and difficult to detect at an early stage, with few effective treatments and low survival rates, making it a major challenge for biomedical research. Indeed, this adenocarcinoma is predicted to become the second leading cause of cancer-related deaths in the United States by 2020 [1, 2]. Current therapies for unresectable tumors, which are the most common ones, include chemotherapy administration based on gemcitabine, folifirinox or nab-paclitaxel, but these are very inefficient and only minimally improve patient survival, such that new therapeutic strategies with improved efficiency are urgently needed. In contrast, other types of less-frequent pancreatic tumors, such as acinar cell carcinomas [3] and pancreatic neuroendocrine tumors [4], are associated with better prognosis and longer survival rates.

Sunitinib is a broad-spectrum receptor tyrosine kinase (RTK) inhibitor whose targets include VEGFR-1,-2,-3, PDGFR-α,-β [5], stem cell factor receptor (c-KIT), Fms-like tyrosine kinase-3 receptor, the glial cell line derived neurotrophic factor receptor (RET) and colony-stimulating factor type 1 receptor (CSF-1R) [5–7]. Sunitinib inhibits endothelial cell proliferation and therefore is considered as an anti-angiogenic drug [8]. However, the expression of sunitinib-targeted RTKs in other stromal cells, as well as in the tumor epithelium, suggests that it could have a more extended mechanism of action with potential multiple effect in these different cells [5, 9, 10]. Sunitinib treatment has given impressive results in neuroendocrine pancreatic tumors, and its use for this pathology was recently approved by the FDA [11, 12]. In contrast, phase II clinical trials to test sunitinib for treating PDA have consistently shown its failure, either when sunitinib was combined with gemcitabine for patients with advanced or metastatic PDA [13] or when it was given as a second-line therapy after gemcitabine failure [14].

The reasons behind this inefficacy of sunitinib treatment in PDA patients are not well understood. Several *in vitro* and *in vivo* reports on pancreatic ductal tumor cell lines and xenografts showed encouraging results, suggesting that sunitinib could potentially be used to improve standard chemotherapeutical treatments [10, 15–20]. *In vivo* sunitinib treatment in transgenic mouse models of pancreatic cancer was ineffective for *k-ras*–driven PDA yet successful for a neuroendocrine preclinical model (RIP-Tag) [21]. Understanding these apparently contradictory results thus requires further studies to elucidate the precise mechanistic roles and potential uses of sunitinib in PDA therapy.

Here, we have tested sunitinib effects in the *Ela-myc* transgenic mouse model of pancreatic cancer, both *in vitro* and *in vivo*. *Ela-myc* mice develop acinar tumors that can undergo acinar-to-ductal metaplasia and progress to ductal adenocarcinomas, mimicking human PDA progression [22–24]. In contrast to xenograft models, transgenic mice can better recapitulate tumor onset, tumor progression and tumor-stroma crosstalk, the latter of which is particularly important in pancreatic cancer. This study using the *Ela-myc* model complements the only one previously published preclinical report of sunitinib in pancreatic cancer that used transgenic mice (with a *k-ras*–based model) [21]. Using the *Ela-myc* model, we were able to analyze sunitinib effects in ductal as well as, for the first time, in acinar tumors. Our *in vivo* and *in vitro* data provide new insights into the therapeutic use of sunitinib for exocrine pancreatic cancer.

## RESULTS

### *Ela-myc* tumors express sunitinib-targeted tyrosine kinase receptors in both stromal and cancer cells

**Figure 1:**
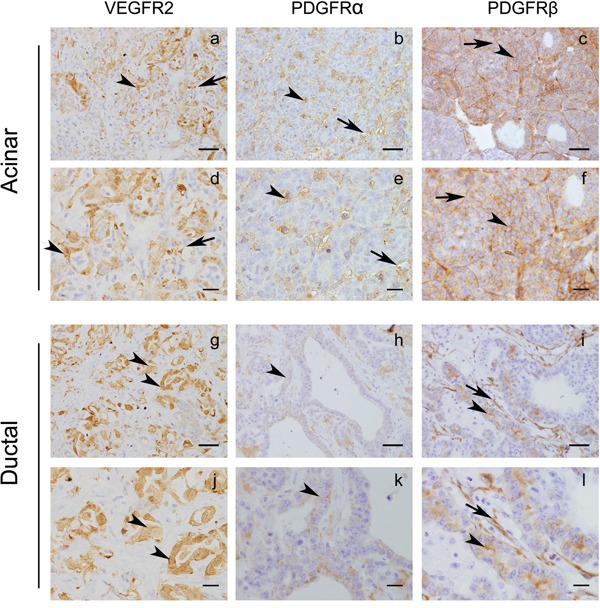
*Ela-myc* tumors express tyrosine kinase receptors that can be targeted by sunitinib Immunohistochemical analyses of *Ela-myc* mice pancreatic tumors were performed to detect tyrosine kinase receptors known to be sunitinib targets, such as VEGFR2, PDGFR-α and PDGFR-β. Scale bars for **a–c.** and **g–i.**, 50 μm; for **d–f.** and **j–l.**, 20 μm.

The *Ela-myc* transgenic mouse model overexpresses the oncogene *c-myc* under the control of the elastase promoter, which leads to acinar pancreatic tumors. Importantly, approximately 50% of these tumors progress to ductal tumors, which are associated with abundant stroma—one of the principle hallmarks of human PDA [22, 23]. Although mutation of *K-Ras* is the most frequent alteration driving pancreatic cancer, *c-Myc* appears to have a key role in PDA development and progression [25–27] and has also been linked with *K-Ras* in this pathology [28, 29]. To test the effects of sunitinib in this model of pancreatic cancer, we first analyzed the expression of sunitinib-targeted RTK in acinar and ductal tumor areas from *Ela-myc* mice (Figure 1). Immunohistochemical analyses showed that VEGFR2, PDGFR-α and PDGFR-β were expressed in pancreatic tumors, both acinar (Figure 1a–1f) and ductal areas (Figure 1g–1l). Importantly, these receptors were expressed not only in blood vessels and tumor stroma (Figure 1, arrows) but also in epithelial cancer cells (Figure 1, arrowheads). These data suggested a potential sunitinib sensitivity of *Ela-myc* tumors, which could be due not only to its well-known anti-angiogenic effect but also to its effects on the RTK that are expressed in pancreatic tumor cells.

### Sunitinib treatment effects on survival, tumor burden, differentiation and necrosis in *Ela-myc* mice

**Figure 2:**
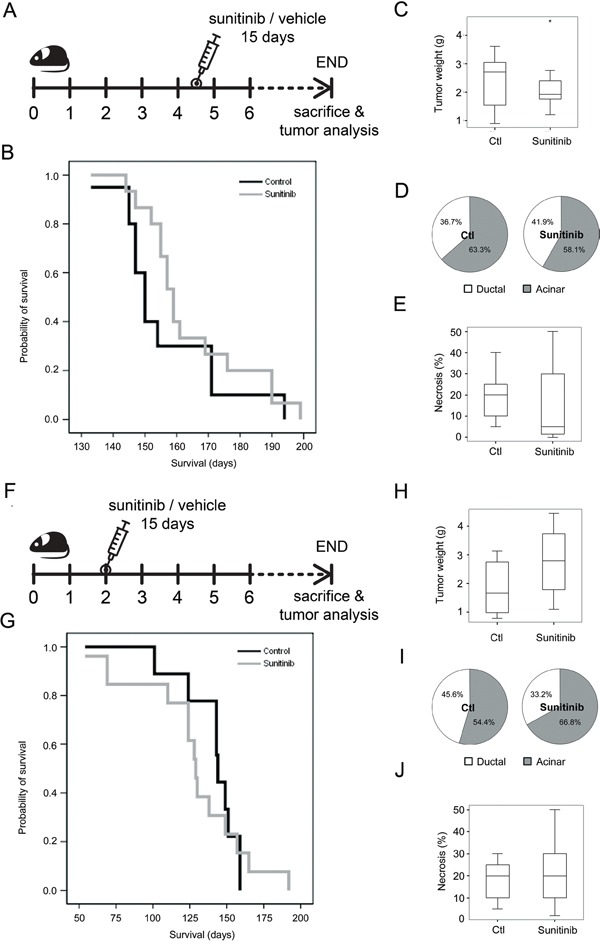
The effects of sunitinib administration on tumor progression and survival in the *Ela-myc* mice model **A.** *Ela-myc* mice with well-established pancreatic tumors (4.5-months old) were treated daily with sunitinib (80 mg/kg; n = 20) or control vehicle (n = 20) for 15 days. Mice were sacrificed at endpoint based on ethical guidelines to study animal survival. **B.** A Kaplan-Meier survival plot showing no significant differences between control or sunitinib-treated animals. **C.** Box-and-whisker plot showing tumor weight at the moment of sacrifice. The asterisk present in the sunitinib treated group is not referring to significance but indicating an extreme outlier. **D.** Pie charts showing the percentage of ductal (white) and acinar (grey) areas in control or sunitinib-treated animal. **E.** Box-and-whisker plot showing tumor necrosis in acinar areas, as determined histopathologically. **F.** Sunitinib administration scheme in 2-month-old *Ela-myc* mice harboring incipient tumors. Similar to treatment of 4.5-month-old mice, no statistically significant differences were observed between treated and control mice regarding survival **G.**, tumor weight **H.**, tumor differentiation **I.** or necrosis **J.**

Next, we analyzed the effects of sunitinib treatment in tumor progression and survival of *Ela-myc* mice. These mice develop pancreatic cancer with 100% penetrance between 2 and 7 months of age. *Ela-myc* mice with advanced tumors (4.5 months old) were treated by oral administration of sunitinib or control vehicle for 15 days. After this treatment, animals were followed until endpoint disease as determined by strict ethical guidelines (Figure 2A). Sunitinib treatment had no significant effect on *Ela-myc* mice survival, as determined by Kaplan-Meier curve analyses (Figure 2B). Similarly, *Ela-myc* tumors from sunitinib-treated mice did not display differences in tumor mass (Figure 2C). Finally, further histopathologic analyses of tumor samples to evaluate tumor differentiation, as determined by the percentage of acinar versus ductal component in each tumor (Figure 2D) and by necrosis (Figure 2E), failed to detect significant differences between sunitinib-treated and control mice. These data indicate that sunitinib therapy is inefficient in well-established pancreatic tumors in the *Ela-myc* cancer model.

To determine if mice at earlier stages of pancreatic tumorigenesis were sensitive to sunitinib therapy, 2-month-old *Ela-myc* mice (which usually have incipient tumors) were treated with sunitinib in a similar manner as the older mice (Figure 2F). However, sunitinib treatment did not significantly affect animal survival (Figure 2G) tumor weight (Figure 2H), differentiation (Figure 2I) or necrosis (Figure 2J).

### Sunitinib treatment effects on tumor angiogenesis in *Ela-myc* mice

**Figure 3:**
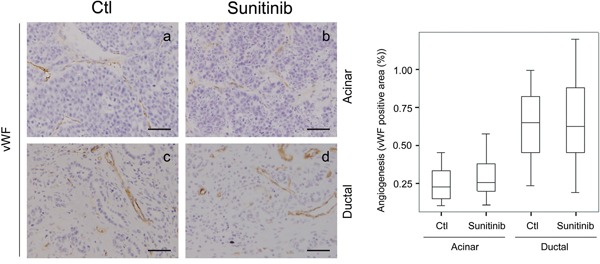
Sunitinib effects on *Ela-myc* pancreatic tumor angiogenesis Tumor angiogenesis was evaluated for pancreatic tumors in control (Ctl) and sunitinib-treated *Ela-myc* mice by von Willebrand Factor (vWF) staining, in both acinar and ductal regions. Quantification of positive areas is shown on the right. Scale bars, 50 μm.

As sunitinib treatment inhibits blood vessel formation in cancer, we tested for an anti-angiogenic effect on *Ela-myc* mice by analyzing the number of blood vessels in pancreatic tumors using von Willebrand factor immunohistochemical staining (Figure 3). *Ela-myc* pancreatic ductal tumor areas displayed higher vascularization as compared to acinar ones (Figure 3; compare a with c), suggesting increased susceptibility to sunitinib anti-angiogenic effects. However, quantification of the number of blood vessels did not show any differences between vehicle-treated (control) and sunitinib-treated mice, either in acinar or ductal tumor areas (Figure 3, right panel). These data indicate that *Ela-myc* pancreatic tumors are not responsive to the anti-angiogenic effects of sunitinib.

### Sunitinib treatment effects on cancer cells in *Ela-myc* mice

**Figure 4:**
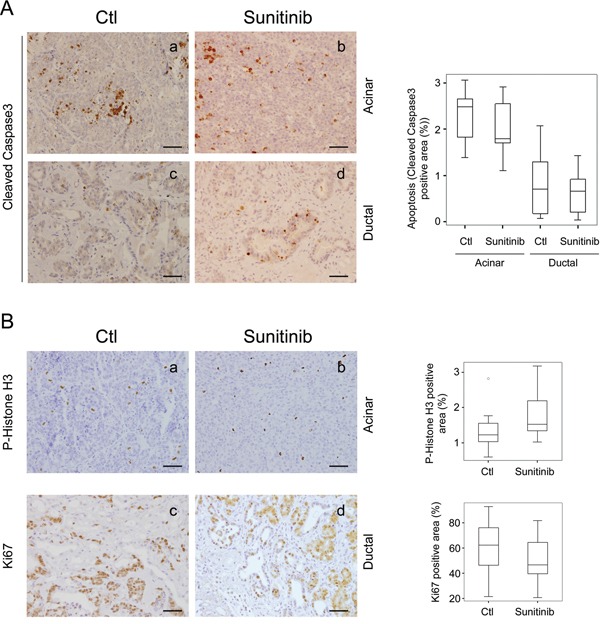
Sunitinib effects in *Ela-myc* pancreatic tumor cell apoptosis and proliferation Tumor cell apoptosis and proliferation was analyzed for pancreatic tumors in control (Ctl) and sunitinib-treated *Ela-myc* mice. **A.** Tumor cell apoptosis was evaluated through cleaved caspase 3 (active form) staining. **B.** Tumor cell proliferation was evaluated through P-Histone H3 staining in acinar lesions, or Ki67 in ductal ones. Box-and-whisker plot quantifications are shown on the right (A, B). Scale bars, 50 μm.

Considering the high expression of sunitinib-targeted RTKs found in *Ela-myc* pancreatic cancer cells (Figure 1), we next aimed to determine whether sunitinib treatment may directly impact cancer cells, by either inducing apoptosis or blocking tumor cell proliferation. To evaluate sunitinib effects on cancer cell apoptosis, we analyzed cleaved caspase 3 staining of both acinar and ductal tumor areas (Figure 4A). As previously reported [30], acinar tumors showed increased cell apoptosis compared to ductal ones (Figure 4A, compare a with c), although quantification of cleaved caspase 3 positive areas showed no significant differences between control and sunitinib-treated animals (Figure 4A, right panel). Subsequently, we tested whether sunitinib treatment could inhibit tumor cell growth by measuring cell proliferation using P-Histone H3 for acinar tumors (Figure 4B, a and b) and Ki67 immunostaining for ductal tumors (Figure 4B, c and d) (see Material and Methods). No significant differences in the proliferative rate of acinar or ductal pancreatic tumor lesions were detected between vehicle-treated and sunitinib-treated animals (Figure 4B, right panels).

Altogether, these data indicate that *in vivo* sunitinib treatment in the *Ela-myc* model has no effect on tumor angiogenesis, cell proliferation or apoptosis, with no impact on tumor progression or animal survival.

### Sunitinib increases cell death and inhibits cell proliferation in *Ela-myc* tumor-derived cell lines

**Figure 5:**
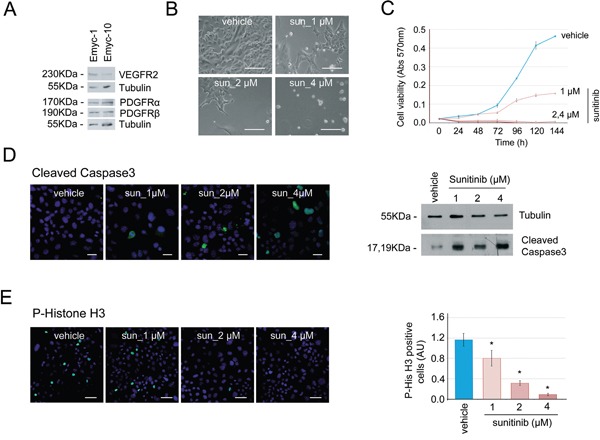
Cells derived from *Ela-myc* pancreatic tumors are sensitive to sunitinib *in vitro* **A.** Analysis by Western blot of the expresssion of the sunitinib-targeted receptors VEGFR2, PDGFR-α and PDGFR–β in two different cell lines derived from *Ela-myc* pancreatic tumors. **B.** Bright field images showing *Emyc-1* cell sensitivity to different doses of sunitinib (1, 2 or 4 μM) *in vitro*, as compared to cells treated with vehicle (DMSO). Scale bars, 100 μm. **C.** MTT experiments were performed to quantify cell viability upon sunitinib treatment (1, 2 or 4 μM) in the *Emyc-1* cell line, observing a dose-dependent effect. **D.** Left, immunofluorescence of cleaved caspase 3, to detect cell apoptosis upon sunitinib treatment (1, 2 or 4 μM). Scale bars, 20 μm. Right, Western blot analysis of the levels of cleaved caspase 3 in *Emyc-1* cell line treated with sunitinib. Tubulin levels are shown as the loading control. **E.** Left, Immunofluorescence of P-Histone H3, to show cell growth arrest upon sunitinib treatment (1, 2 or 4 μM) in *Emyc-1* cells. Scale bars, 50 μm. Right, Bar plots showing quantification of P-Histone H3 immunofluorescence experiments on the right. *p < 0.05 (Student's t-test).

Fibrotic stroma is one of the major hallmarks of pancreatic cancer, and its role in hindering efficient drug delivery and promoting therapy resistance is well known [31]. To determine if the lack of effects *in vivo* of sunitinib treatment in *Ela-myc* mice could be due to impaired drug delivery because of this pancreatic fibrotic barrier, we tested sunitinib *in vitro* on two distinct *Ela-myc* pancreatic tumor–derived cell lines (*Emyc-1* and *Emyc-10*). Similar to *Ela-myc* pancreatic tumors *in vivo*, these cells expressed the sunitinib-targeted RTKs VEGFR2, PDGFR-α and PDGFR-β (Figure 5A). Next, we tested the susceptibility of these cells *in vitro* to different doses of sunitinib. *Emyc-1* cells treated with sunitinib (1 to 4 μM) showed impaired viability as compared to vehicle-treated cells, as determined by bright field microscopy (Figure 5B). Kinetic quantification of cell viability by MTT detection clearly showed that *in vitro* sunitinib treatment of *Emyc-1* cells was strongly cytotoxic, in a specific and dose-dependent manner, as compared to vehicle treatment (Figure 5C). We then analyzed whether the sunitinib-induced cytotoxicity was a consequence of increased apoptosis, decreased proliferation, or both, by detecting the active form of caspase 3 and P-Histone H3, respectively. *In vitro* treatment of *Emyc-1* cells with sunitinib resulted in increased apoptosis, shown by immunofluorescence and Western blot detection of cleaved caspase 3 (Figure 5D), and in a dose-dependent reduction of cell proliferation, shown by immunofluorescence staining and quantification of P-Histone H3 (Figure 5E). Similar dose-dependent susceptibility to sunitinib treatment was observed in *Emyc-10* cells (data not shown).

Thus, *in vitro*, sunitinib increases cell death and reduces cell proliferation of *Ela-myc*–derived pancreatic cancer cell lines. This suggests that the lack of effects observed *in vivo* may be due to impaired drug delivery to the pancreatic tumor, rather than cell insensitivity to the drug.

### Sunitinib treatment affects subcutaneous tumors established from *Ela-myc* tumor-derived cell lines

**Figure 6:**
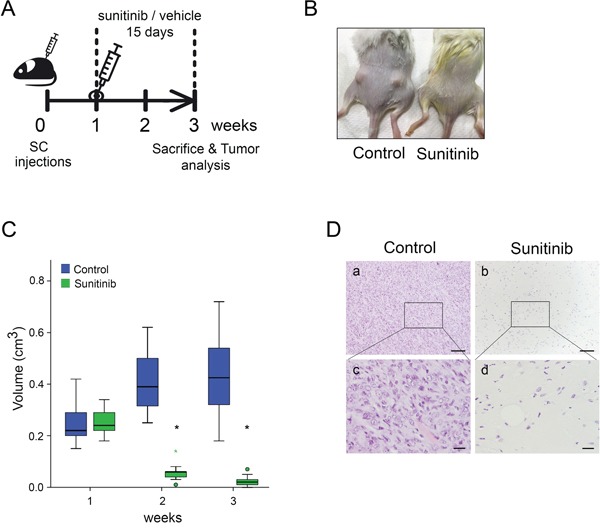
*Ela-myc*–derived subcutaneous tumors are sensitive to sunitinib **A.** Schematic representation of the design of the *in vivo* subcutaneous experiment. SCID Beige animals were injected with 3 × 10^6^ *Emyc-1* cells per flank and, when tumors reached 0.5 cm^2^ (at 1 week), animals were orally administered with sunitinib or control vehicle for 15 days (10 mice, thus 20 tumors per condition). After treatment, tumors were collected and analysed. **B.** Representative images of mice with *Ela-myc*–derived subcutaneous tumors after 15 days of treatment with vehicle or sunitinib. **C.** Box plot showing tumor size distribution of sunitinib- (green) or vehicle-treated (blue) animals before treatment (week 1), during (week 2) and after (week 3) drug delivery. Green circles label outliers and green asterisks extreme outliers. *p < 0.05 (Mann Whitney test). **D.** H&E staining of *Ela-myc* allografts after treatment with control vehicle (panels a, c) or sunitinib (panels b, d). Scale bars, 100 μm (a, b), 20 μm (c, d).

We next tested the efficiency of sunitinib *in vivo* on tumors from *Emyc-1* cells injected subcutaneously in the dorsal flanks of immunosuppressed mice. Once tumors reached 0.5 cm^2^, animals were orally treated with sunitinib or control vehicle, and evaluated for drug effects. After a daily treatment with sunitinib or vehicle for 15 days mice were sacrificed and tumors analyzed (Figure 6A). Interestingly, sunitinib administration caused complete tumor regression (Figure 6B, 6C). H&E and histopathological analysis revealed subcutaneous tumors only in mice treated with control vehicle, whereas in mice treated with sunitinib we only observed an inflammatory abscess with no presence of tumor cells (Figure 6D).

These data suggest that the ineffectiveness of sunitinib treatment in *Ela-myc* transgenic mice was due to the pancreatic tumor localization. This most likely due to its abundant desmoplastic stroma, as tumors derived from this model in a non-pancreatic niche were highly responsive to the drug *in vivo*.

## DISCUSSION

**Figure 7:**
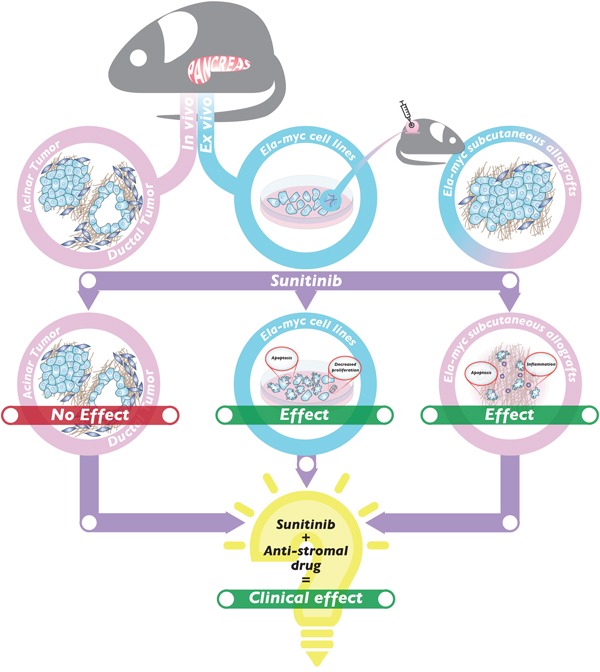
Working model of the *in vivo* and *in vitro* effects of sunitinib in the *Ela-myc* mouse model Sunitinib treatment of *Ela-myc* pancreatic acinar and ductal tumors does not show any significant effect *in vivo* (left). In contrast, *Ela-myc*–derived tumor cell lines are highly sensitive to sunitinib *in vitro*, with increased apoptosis and decreased proliferation (center). Importantly, subcutaneous injection of these cells in SCID Beige mice forms tumors that are highly sensitive to sunitinib treatment (right). These data may help understand sunitinib failure for PDA patients and suggest that a combination of sunitinib and an anti-stromal drug may be a promising strategy for successfully treating this pathology in the clinics.

Gemcitabine was already in use as a first-line PDA therapy by 1997, but without high success rates [32]. Patient survival has slightly improved since then through the use of novel chemotherapy combinations [33, 34], but these new treatments are still unable to reach one year as patient median overall survival, leaving PDA as one of the tumors with the worst prognosis. Thus, new effective therapies are urgently needed to treat this pathology. The multi-target inhibitor of RTKs, sunitinib, recently emerged as a possible hope for PDA therapy. However, although sunitinib therapy has improved treatment of tumors like renal cell cancer and gastrointestinal stromal tumors, it has not led to improvements for PDA patients [13, 14]. Using a preclinical mouse model of pancreatic cancer, we now provide insights to the failure of sunitinib treatment in this pathology as well as potential ways in which this can be overcome. Specifically, we have reported a preclinical study that addresses the impact of sunitinib in pancreatic ductal and acinar tumors using a transgenic mouse model of pancreatic cancer. We found no significant differences on tumor progression or hallmarks or on survival between sunitinib-treated animals and control ones (Figure 7). However, tumor derived cell lines did respond to sunitinib *in vitro* in a dose-dependent manner. Further, allografts from subcutaneous injections of *Ela-myc* tumor–derived cells regressed after sunitinib treatment, suggesting that the complex tumor microenvironment present in the pancreatic niche impairs sunitinib effects in the *Ela-myc* model (Figure 7).

One of the best characterized effects of sunitinib as an anti-cancer therapy is its ability to inhibit angiogenesis by targeting VEGFR on tumor endothelial cells. As angiogenesis is a key contributor to tumor progression and metastasis in most solid tumors, a large number of preclinical and clinical studies have focused on targeting tumor vasculature as an anti-cancer therapy [35]. However, tumor vessels are sparse and poorly functional in PDA, and whether angiogenesis is important for its progression is controversial. On the one hand, hypovascularization of PDA should render these tumors very dependent on this limited vascular network and therefore highly sensitive to anti-angiogenic therapies. Accordingly, several preclinical studies have gathered data showing the benefits of anti-angiogenic treatments in PDA [15, 36, 37]. On the other hand, recent data have shown that increasing blood perfusion and normalizing the vasculature development favor drug delivery and survival in PDA animal models [38]. Moreover, in PDA patients, clinical trials using anti-angiogenic drugs have repeatedly failed [39–41].

An important advantage of sunitinib as compared to standard anti-angiogenic treatments is that it exerts additional anti-tumor effects [42, 43]. Thus, sunitinib can impair tumor desmoplasia by targeting PDGF-dependent fibroblasts proliferation [44] and can block tumor growth by inhibiting several RTKs in cancer cells [5, 6, 42]. Indeed, sunitinib has shown excellent results both in preclinical and clinical settings for different tumors [5, 8, 42, 45, 46] and has gained FDA approval for several neoplasms [11, 47].

However, despite its multi-bullet targeting, sunitinib has not proven effective for PDA. In preclinical mouse models using pancreatic xenografts, the overall survival rate was not improved by treatment of sunitinib alone [10] but was improved when sunitinib was combined with chemotherapy [10, 15–20], radiotherapy [19, 48] or miRNA strategies [49]. In contrast, sunitinib failed to increase survival rates in either the *k-ras* or the *c-myc* genetically engineered mouse models ([21] and this manuscript), even in combination with gemcitabine [21]. Here, it is important to note that *k-ras–* and *c-myc*–driven pancreatic cancer mouse models, in contrast to xenograft models, recapitulate histopathology and progression of human pancreatic cancer, including acinar-to-ductal metaplasia and a strong desmoplastic reaction [23, 38, 50]. In fact, fibrotic stroma has been reported to act as a mechanical barrier hampering chemotherapy delivery in PDA [31], which could explain the lack of response after sunitinib treatment in these transgenic models. In support of this hypothesis, we found that even though sunitinib treatment had no effects on tumor volume or histopathological hallmarks in *Ela-myc* mice *in vivo*, cancer cells derived from these tumors were highly sensitive to the drug *in vitro*. Further, after injecting cells derived from these tumors into a non-pancreatic niche in the mouse, sunitinib was highly effective *in vivo*, suggesting that sunitinib exerts its anti-cancer effects if it can efficiently reach the tumor (Figure 7). Importantly, therapeutic targeting of the tumor stroma has given encouraging results in preclinical studies and is now being tested in clinical trials [38, 51, 52]. Moreover, one of the few improvements in managing human PDA in the last 20 years has been by treating with a combination of gemcitabine and nab-paclitaxel, which compromises the stromal architecture to increase perfusion [34]. Preliminary data from mice xenograft PDA models combining nab-paclitaxel with sunitinib are positive [17], although further future preclinical experiments using sunitinib in combination with stromal disrupting drugs are required to better understand the anti-tumor effects of sunitinib in PDA therapy.

In agreement with a lack of sunitinib effectiveness in the *k-ras* and *c-myc* genetically engineered mouse models ([21] and this manuscript), sunitinib failed in human PDA treatments when administered as a second-line therapy [14] or in combination with gemcitabine [13]. These data reinforce the idea that xenograft animal models have a low predictive value as compared to genetically engineered models in preclinical studies [53] and highlight the necessity of obtaining extensive preclinical data before moving to patients. In fact, an important meta-analysis of preclinical studies with sunitinib showed that only 2% of these had used genetically engineered mouse models [54]. These data encouraged us to replenish the lack of preclinical information with transgenic models for PDA pathology, using *Ela-myc* mice as a different transgenic pancreatic tumor model from the previously published *k-ras* model [21].

The molecular complexity of PDA tumors, which are genetically heterogenous, can explain why sunitinib gave positive results in maintenance therapy in metastatic PDA that had achieved disease control with first-line therapy (a very rare subset of PDA patients) [55]. Likewise, a case report has documented an impressive extended survival after treating with gemcitabine and sunitinib [56]. Thus, the lack of patient selection in published clinical trials may have underestimated the sunitinib potential for PDA treatment, highlighting the importance of personalized medicine in identifying patients who can benefit from sunitinib.

Finally, even though PDA is the most common type of pancreatic cancer, there are patients with other less frequent pancreatic neoplasms who respond poorly to conventional treatments and who might benefit from sunitinib therapy. For instance, patients with pancreatic neuroendocrine tumors, which represent around 1.3% of pancreatic cancers, have shown a very good response to sunitinib in preclinical models [21, 43, 57] and clinical trials [11, 58, 59]. Indeed, sunitinib has been recently approved by the FDA for the treatment of these tumors [11]. These differences in sunitinib response between neuroendocrine and PDA tumors might be explained by the high vascularization of the former and/or by the dense desmoplasic reaction that hampers drug delivery in PDA. Another uncommon pancreatic cancer is acinar cell carcinoma, which accounts for 1% to 2% of all pancreatic tumors in adults and up to 15% in infancy. Surgical resection is the only chance of cure for acinar cancer, and there is a lack of systemic successful therapies for advanced non-resectable tumors [60]. Research on acinar cancer treatment has been handicapped by the low number of acinar cell lines available, which has limited the *in vitro* laboratory tools, compromised the use of xenografts *in vivo* and resulted in a lack of preclinical studies addressing acinar tumor therapy. Moreover, as this pancreatic cancer type is very rare [61], clinical trials are unfeasible, limiting studies to small sample sizes and case reports. The transgenic *Ela-myc* mouse model, which develops mixed acinar and ductal tumors, now provides the first tool for studying sunitinib treatment of pancreatic acinar cell carcinomas. Our data show that, similar to PDA tumors, acinar tumors do not respond *in vivo* to sunitinib in this preclinical model. Unfortunately, we cannot test *in vitro* sunitinib effects on acinar cells derived from *Ela-myc* tumors, as these cells undergo acinar-to-ductal metaplasia in culture. Therefore, whether this lack of sunitinib efficacy in *Ela-myc* acinar tumors *in vivo* is due to inefficient drug delivery into the tumor, or to insensitivity of acinar cells to the drug, requires further investigation.

The data reported here fit with previous preclinical results with sunitinib in PDA transgenic models and, more importantly, with unselected clinical trials for this pathology. Overall, these results stress the importance of using genetically engineered mouse models rather than xenograft mouse models in preclinical studies. We hypothesize that combining sunitinib with agents targeting the stroma (rather than with the conventional gemcitabine) and segregating patient cohorts using personalized medicine could result in the successful use of this drug for PDA patients.

## MATERIALS AND METHODS

### Mice and sunitinib treatments

Animal procedures were approved by the PRBB Ethical Committee for Animal Experimentation. Founder *Ela-myc* mice (C57Bl/6 genetic background) were kindly provided by E. Sandgren (University of Wisconsin-Madison, WI). Mice were housed and fed *ad libitum* as previously described [22, 62]. Genotyping primers: c-*myc* (5′-CAC CGC CTA CAT CCT GTC CAT TCA AGC-3′ and 5′-TTA GGA CAA GGC TGG TGG GCA CTG-3′), resulting in a 200 bp band. SCID Beige mice were obtained from the PRBB Animal Facility. *Emyc-1* cells (3 × 10^6^) were injected subcutaneously in the two dorsal lateral flanks of 20 SCID Beige mice. Sunitinib (kindly provided by Pfizer) was prepared in a vehicle solution (0.5% carboxymethylcellulose, 1.8% NaCl, 0.4% Tween-80, 0.9% benzyl alcohol) and kept in the dark. Mice (n = 10 for each condition) were treated for 15 days by oral administration daily with 80 mg/Kg of sunitinib malate (corresponding to 60 mg/Kg of the active ingredient) or vehicle solution, according to the manufacturer instructions. *Ela-myc* mice were treated when they were 2- or 4.5-months old, for incipient or advanced tumors, respectively; SCID Beige mice were treated when tumors reached 0.5 cm^2^.

*Ela-myc* mice were sacrificed when tumors compromised animal well-being, as judged by the protocol of Morton and Griffiths [63]. SCID Beige mice were treated with sunitinib or the control vehicle for 15 days and tumor size in all mice was measured weekly with a caliper. Mice were sacrificed, tumors were weighed, fixed in buffered formalin for 24 h, dehydrated and embedded in paraffin.

### Histopathology and immunohistochemistry

For histopathological analysis, tumor sections from mice treated with sunitinib or control vehicle were stained with H&E and evaluated by two expert pathologists (M.I. and J.M-C.) to determine acinar ductal differentiation and the necrotic index [30, 62].

For immunohistochemistry, 5-μm sections from formalin-fixed paraffin-embedded tissue blocks were deparaffined and boiled with 0.01 M citrate buffer (pH 6.0) at 120°C for 10 min in a pressure cooker. Endogenous peroxidase activity was quenched with 3% H_2_O_2_, and samples were blocked in PBS with 1% BSA. Primary antibodies were added overnight at 4°C. Antibodies used for specific tissue immunostaining included anti-VEGFR2 (Cell Signaling Technology), anti-PDGFR-α (R&D) anti-PDGFR-β (Cell Signaling Technology), anti-vWF (Neomarkers), anti-cleaved caspase 3 (R&D systems) anti-P-Histone H3 (Ser10) (Millipore) and anti-Ki67 (Novo Castra). Negative controls were performed with pre-immune rabbit serum. As secondary antibodies, peroxidase-conjugated (Envision+, Dako) anti-rabbit Ig reagents were used. Reactions were developed using 3,3′-diaminobenzidine (DAB) as chromogenic substrate (Dako). Sections were counterstained with hematoxylin, dehydrated and mounted. An Olympus BX61 microscope was used for visualization, and images were acquired using CellSens software.

For quantification, 10 images were acquired at 10× for each tumor and analyzed with ImageJ software. To quantify angiogenesis and apoptosis, the area stained positive for vWF or active caspase 3, as indicated, was related to total area. Due to intrinsic differences in their proliferation, ductal and acinar cells were immunostained with Ki67 or P-Histone H3, respectively, as previously described [62]. For quantification, the percentage of Ki67 (in acinar regions) or P-Histone H3 positive cells (in ductal regions) were obtained by relating the area corresponding of nuclei positive for Ki67 or P-Histone H3 to the total nuclei area (positive for hematoxylin).

### Cell lines

*Emyc-1* and *Emyc-10* cell lines were obtained from *Ela-myc* tumors as previously described [64, 65]. Cells were cultured in DMEM supplemented with 10% FBS at 5% CO_2_ and 37°C.

### Western blots

Whole cell extracts were prepared with Laemmli buffer, and samples were boiled at 95°C for 5 min. Proteins were resolved by SDS-PAGE and transferred to nitrocellulose filters for Western blot analysis. The primary antibodies used were: anti-VEGFR2 (Cell Signaling Technology), anti-PDGFR-α and anti-PDGFR-β (Santa Cruz), anti-cleaved caspase 3 (Cell Signaling Technology), anti-P-Histone H3 (Ser10) (Millipore) and anti-tubulin (Sigma). Specie-specific secondary antibodies conjugated to HRP (Dako) and ECL detection (Amersham) were used for band visualization.

### Cell viability

*In vitro* cell viability of *Emyc-1* cells was assessed with 3-(4,5-dimethylthiazol-2-yl)-2,5-diphenyltetrazolium bromide staining (MTT). Cells were seeded in 96-well plates in quintuplicate at a density of 1000 cells per well and grown in 2% FBS with the sunitinib vehicle (DMSO) or in the presence of sunitinib in increasing concentrations (at 1, 2, or 4 μM). Cell viability was measured daily by incubating cells with MTT (1 mg/mL) for 4h. The formazan precipitate was extracted with DMSO:isopropanol (1:4), and absorbance was taken at 570 nm on a multiwell-plate reader.

### Immunofluorescence to detect apoptosis and proliferation

Cells seeded over sterile coverslips and grown in 10% FBS were fixed in 4% PFA. Cells were permeabilized with 0.2% Triton in PBS and blocked with 5% BSA, 0.1% Tween-20. Coverslips were incubated overnight with rabbit anti-P-Histone H3 (Ser10) (Millipore) to determine proliferation or with rabbit anti-cleaved caspase 3 (R&D) to determine apoptosis. Negative controls were incubated with an irrelevant rabbit IgG (data not shown). An anti-rabbit Alexa Fluor 488 (Invitrogen) was used as the secondary antibody. Coverslips were mounted with DAPI Fluoromont-G (Southern Biotech), and IF was detected with an Olympus BX61 Microscope. Ten fields were quantified per coverslip at 10×. The number of P-Histone H3 positive cells was manually counted and compared to the total number of cells (DAPI positive) for each experiment, which was quantified using an ImageJ macro developed in the CRG Advanced Light Microscopy Unit facility (CRG, Barcelona).

### Statistical analysis

Statistical analyses were performed with SPSS version 12.0. Statistical significance cut-off was considered as *p* < 0.05. Kaplan-Meier analyses were used to establish survival curves, and comparisons were made using the log-rank test. The Student's t-test was used with normally distributed data (*in vitro*) and the Mann-Whitney test was used in other occasions (*in vivo*).
